# STELO: A New Modular Robotic Gait Device for Acquired Brain Injury—Exploring Its Usability

**DOI:** 10.3390/s24010198

**Published:** 2023-12-29

**Authors:** Carlos Cumplido-Trasmonte, Eva Barquín-Santos, María Dolores Gor-García-Fogeda, Alberto Plaza-Flores, David García-Varela, Leticia Ibáñez-Herrán, Carlos González-Alted, Paola Díaz-Valles, Cristina López-Pascua, Arantxa Castrillo-Calvillo, Francisco Molina-Rueda, Roemi Fernandez, Elena Garcia-Armada

**Affiliations:** 1 International Doctoral School, Rey Juan Carlos University, 28922 Madrid, Spain; carlos.cumplido@marsibionics.com; 2 Marsi Bionics SL, 28521 Madrid, Spain; eva.barquin@marsibionics.com (E.B.-S.); alberto.plaza@marsibionics.com (A.P.-F.); david.garcia@marsibionics.com (D.G.-V.); leticia.ibanez@marsibionics.com (L.I.-H.); elena.garcia@marsibionics.com (E.G.-A.); 3 Department of Physical Therapy, Physical Medicine and Rehabilitation, Faculty of Health Sciences, Rey Juan Carlos University, 28922 Madrid, Spain; francisco.molina@urjc.es; 4 Spanish National Reference Centre for Brain Injury (CEADAC), 28034 Madrid, Spain; cgonzaalted@imserso.es (C.G.-A.); paoladiazvalles@gmail.com (P.D.-V.); 5 Centro Lescer, 28035 Madrid, Spain; clopez@centrolescer.org (C.L.-P.); acastrillo@centrolescer.org (A.C.-C.); 6 Centre for Automation and Robotics (CAR), CSIC-UPM, Ctra. Campo Real km 0.2–La Poveda-Arganda del Rey, 28500 Madrid, Spain

**Keywords:** robotics, gait, neurology, ABI, modular

## Abstract

In recent years, the prevalence of acquired brain injury (ABI) has been on the rise, leading to impaired gait functionality in affected individuals. Traditional gait exoskeletons are typically rigid and bilateral and lack adaptability. To address this, the STELO, a pioneering modular gait-assistive device, was developed. This device can be externally configured with joint modules to cater to the diverse impairments of each patient, aiming to enhance adaptability and efficiency. This study aims to assess the safety and usability of the initial functional modular prototype, STELO, in a sample of 14 ABI-diagnosed participants. Adverse events, device adjustment assistance and time, and gait performance were evaluated during three sessions of device use. The results revealed that STELO was safe, with no serious adverse events reported. The need for assistance and time required for device adjustment decreased progressively over the sessions. Although there was no significant improvement in walking speed observed after three sessions of using STELO, participants and therapists reported satisfactory levels of comfort and usability in questionnaires. Overall, this study demonstrates that the STELO modular device offers a safe and adaptable solution for individuals with ABI, with positive user and therapist feedback.

## 1. Introduction

Acquired brain injury (ABI) refers to any damage that occurs to the brain after birth resulting from traumatic or non-traumatic causes [1]. The incidence and prevalence of ABI vary worldwide, but it is a significant public health concern affecting individuals of all ages [2]. Incidence is influenced by various factors such as the occurrence of accidents, violence, strokes, tumors, and other medical conditions [2,3].

ABI can result in muscle weakness, impaired coordination, spasticity, and sensory deficits, all of which can affect the ability to walk, causing gait abnormalities such as ataxia, dystonia, and apraxia [4]. These physical challenges can significantly impact the independence and quality of life for individuals with ABI. Walking difficulties may require the use of assistive devices like canes, walkers, or wheelchairs to maintain mobility [1]. Rehabilitation programs play a crucial role in addressing gait impairments by focusing on improving muscle strength, balance, coordination, and restoring functional abilities [5].

In addition to physical implications, ABI can also have cognitive and emotional effects, which may further complicate the management of gait disturbances [6]. Cognitive deficits such as memory problems, attention difficulties, and executive dysfunction can impact the ability to plan and initiate movements during walking [6]. Emotional changes, including depression, anxiety, and mood swings, can also influence motivation and engagement in rehabilitation programs aimed at improving gait [7].

The development of innovative technologies, such as exoskeletons, has opened new avenues for assisting patients with ABI in regaining mobility and enhancing their overall quality of life [8]. Robotic gait rehabilitation offers precise and repetitive training in an engaging environment. It reduces the physical burden on therapists while providing objective assessments of patient progress [9]. Compared with traditional therapy, robotic gait rehabilitation allows for controlled and intensive training, promoting motor recovery and optimizing treatment outcomes [10].

Currently, the design of robotic exoskeletons is rigid and bilateral and does not allow for adaptations to patient characteristics and needs, resulting in poor usability [11]. Poor usability is due to several crucial factors that are common in most current devices. They are often reported as difficult to wear autonomously (primarily due to their considerable weight and size) and as a “monolithic” structure that creates challenges in donning and doffing, transportation, and overall device handling [11]. As a consequence, a new prototype of a modular and intrinsically intelligent configuration has been tested based on the use of neural network-based control algorithms [12]. With this innovative technology, named STELO, it is possible to externally configure the device according to the user´s functional needs. This system offers the flexibility to be assembled in specific configurations that can address the specific gait deficits of individual patients [13]. Therapists can choose and configure the necessary modules based on the functional capacity of each patient. This customization allows for a tailored approach that optimizes the effectiveness of the robotic gait device in meeting the specific needs of each user. Therefore, it is a completely (functionally and physically) modular prototype that challenges the design of current exoskeletons [14].

Although other modular prototypes have been studied regarding spinal cord injury [15], to the best of our knowledge, there is no complete modular device that has been tested in individuals with ABI. Therefore, the aim of this study is to evaluate STELO´s usability in a clinical environment for individuals diagnosed with ABI. The term “usability” can be considered as the “quality of use” and defined as “the extent to which a system, product, or service can be used by users to achieve specific goals with effectiveness, efficiency, and satisfaction in a specific context” (ISO 9241-210:2009) [16]. Thus, usability in the present study is understood as a combination of safety, effectiveness, and satisfaction.

## 2. Materials and Methods

This single group open label intervention trial was conducted at the Spanish National Reference Centre (CEADAC, Madrid, Spain) and Centro Lescer (Madrid, Spain). The Standard Protocol Items: Recommendations of Interventional Trials (SPIRIT) guidelines [17] were followed to ensure this study’s quality. This study was performed in accordance with the Declaration of Helsinki [18], and approval was obtained by University Hospital of Getafe and the Spanish Drug and Medical Devices Agency (reference 961/21/EC-R) prior to the start of the study. The clinical trial was registered on Clinical Trials.gov: NCT05265377 and was conducted in March and April of 2023.

### 2.1. Participants

This clinical study focused on individuals with ABI. Inclusion criteria for participant eligibility were as follows: (1) age between 18 and 85 years, (2) weight below 100 kg, (3) height ranging from 150 to 190 cm, (4) hip width between 30 and 45 cm, (5) distance from the hip joint center to the knee joint center 36–50 cm, (6) distance from the joint center to the ground 43.5–59.5 cm, (7) European shoe size between 36 and 45, (8) ability to understand and follow simple commands, and (9) a functional ambulation category (FAC) [19] score below or equal to 4.

Exclusion criteria for participation in this study were the following: (1) lower limb spasticity score of 4 on the modified Ashworth scale (MAS) [20], (2) skin alterations in areas of contact with the device, (3) planned surgical intervention during this study, (4) two or more osteoporotic fractures in the lower limbs within the past 2 years, (5) exercise intolerance, (6) limb and/or spine surgery within the 3 months prior to the start of this study, and (7) psychiatric disorders that may interfere with proper device use or study participation, such as impulsivity or inability to understand simple commands.

Written informed consent was obtained from all participants prior to their inclusion in the study.

### 2.2. Device

**Figure 1:**
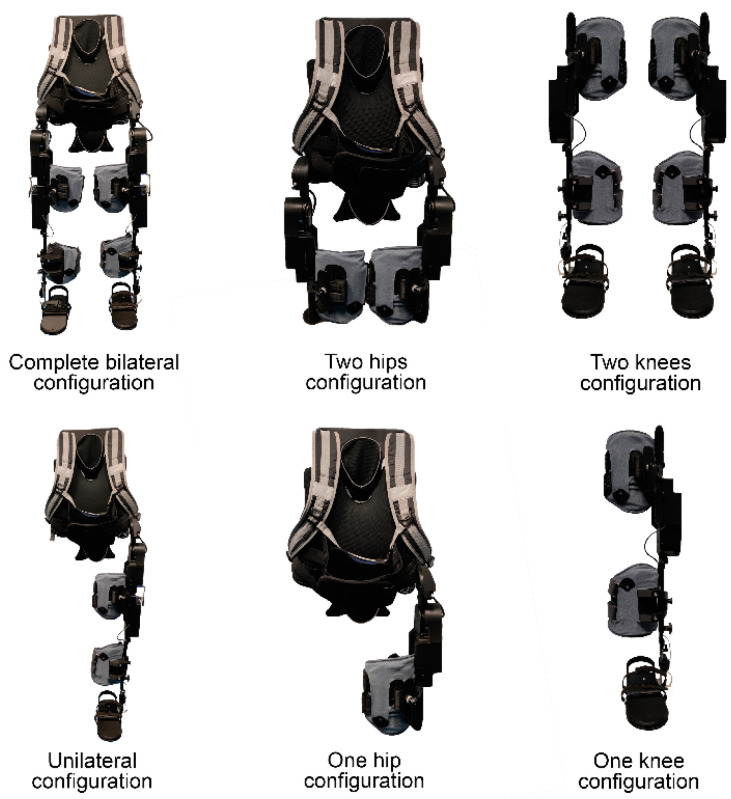
Six different module configurations of STELO.

STELO is a modular overground robotic exoskeleton (Figure 1). This device complements the motor action of users by amplifying lower limb strength and mobility. Its main innovation is functional modular capability, which allows the therapist to select the actuated joints according to the patient’s functional needs by mechanically and electronically connecting/disconnecting the actuation modules. This functioning is based on a decentralized control architecture that does not require a main controller for the generation and coordination of the trajectories of the actuated joints as it is replaced by a set of local controllers each associated with a module. These actuation modules correspond to the joints of both hips and knees in the sagittal plane.

The actuation of the STELO exoskeleton is based on a reduced version of the ARES technology [21] that allows to modify the stiffness of the actuator and measure the interaction forces detected in the elastic elements placed in series with the actuator output. Using this mechanism, the power transmission is smoother and compatible with the symptoms of neurological pathologies, such as spasms, tremors, or spasticity. Additionally, this system allows the detection of the force and resistance exerted by the user, which is essential when the patient retains some residual strength to perform control algorithms based on the detection of movement intention and assist as needed.

On the other hand, the exoskeleton modules have been designed using the modular technology of the Marsi active knee (MAK) device [22,23], already certified as electro-medical equipment. Nevertheless, some adaptations regarding the electric motors and gears have been created to provide sufficient power to assist flexion and extension movements during walking, sitting, and standing at each joint. The connection of these actuation modules allows the device to be used in multiple configurations depending on the characteristics of the patient. The motion in each module/articulation of the device is powered by an interchangeable battery placed on the backpack of the torso.

The proposed exoskeleton consists of the following modular components: a trunk structure, two hip modules, two knee modules, two thigh braces, and two calf braces. The trunk structure consists of a backpack with shoulder straps and an abdominal elastic belt that secures the patient’s trunk. This structure is only necessary when using any of the hip modules. The hip modules can be attached either to the trunk structure, using a guide system, or to the corresponding knee module through the thigh supports. The knee modules also require the use of the calf brace on the structure that runs parallel to the leg down to the feet to transmit the power of the actuator.

The actuator, based on the ARES mechanism, includes springs to smoothly transmit forces, absorb shocks, and provide better behavior in cases of patients suffering from spasticity. The decentralized architecture is based on a 32-bit microcontroller in each module, which takes care of the perception system related to that joint, communicates with the other modules of the selected configuration through a multimaster protocol, develops the control strategy, and commands the trajectory to the actuator.

Therefore, in the developed architecture, all the actuation modules of the exoskeleton are at the same hierarchical level. The perception system of each module obtains the in-formation of the angular position and angular velocity through an encoder, the orientation of the module with respect to the ground through an inertial measurement unit (IMU), and the torque applied by the actuator through the spring deformation measurement of the SEA. In addition, ground reaction force (GRF) sensors placed in the platforms provide this information to all modules.

The generation of the coordinated trajectory for the distributed system is based on an algorithm that emulates the biological behavior of the automatisms related with the walking process, named as the central pattern generator (CPG) [12]. This algorithm uses adaptive frequency oscillators that have been previously trained to replicate the angular trajectory of each joint and, related to the other modules, to keep the synchronization during the movement. Using this distributed control algorithm, the computational load can be shared among the different joints of the device that are connected. Further details on the design can be found in [12].

### 2.3. Intervention Procedure

The training protocol involved three 30 min sessions of robotic gait training. During the first session, all participants used the device in a complete bilateral configuration to test it. In the subsequent sessions, the configuration of the modules was adjusted according to the specific impairment of each participant. These sessions took place on non-consecutive days over the course of one week to avoid fatigue. The training was conducted indoors in rehabilitation settings on a smooth and level surface. Depending on the participant’s functional ambulation level, external support was provided, which could include the use of a walker, crutches, or physical contact with a physiotherapist.

### 2.4. Outcome Measures

During the first session, participant demographics, including age and anthropometric measurements, were collected. These measurements, such as weight, height, hip width, femur length, lower limb length, thigh circumference, calf circumference, abdominal circumference, torso width, and shoe size, were gathered to achieve an appropriate fit to the participant’s body.

To assess safety, adverse events (AEs) that occurred while using the device were recorded. Potential AEs included patient and/or therapist falls, a 3-point increase in patient- and/or therapist-reported pain using the visual analogue scale (VAS) [24] at the end of the session, an increase in fatigue reported by the patient and/or therapist using the Borg scale [25] that led to session termination, and any physical injury caused by the device.

Participants’ vital signs, spasticity, and skin integrity were assessed before and after each exoskeleton session. This was performed to compare the initial and final values after exoskeleton use and evaluate if there were increases in lower limb spasticity or vital signs outside the normal ranges described in Alghatani et al. [26]. Vital signs included heart rate (beats per minute), systolic and diastolic blood pressure (mmHg), and oxygen saturation (% SpO2). These measurements were taken using the PD-900 PRO^®^ vital signs monitor (Creative Medical^®^, Shenzen, China). Lower limb spasticity was evaluated using the MAS [20] for the muscles at hips, knees, and ankles.

Furthermore, any serious adverse events (SAEs) as defined by the United States Food and Drug Administration (FDA)—events causing death, life-threatening situations, hospitalization, disability, congenital anomalies, or requiring intervention to prevent permanent impairment or damage—were also recorded [27].

To assess usability, we measured the time required to put the device on participants, as well as the level of assistance needed to complete this task (ranging from one person to three people). These measurements are considered crucial because an excessively long time to don the device and a need for excessive assistance can indicate poor usability. This is significant, since a challenging adjustment process or one that requires significant help can lead to discomfort and unnecessary delays in its use, negatively impacting the user experience.

Functional status assessments [28], including functional ambulation categories (FAC) [19] and timed up and go (TUG) [29], were conducted during the first session without the device. During subsequent sessions, the 6 min walking test (6 mwt) [30] and 10-meter walk test (10 mwt) were performed while wearing the robotic device.

Upon completion of the three sessions, all participants filled out the Quebec user evaluation of satisfaction with assistive technology (QUEST 2.0) scale [31] and the participant satisfaction questionnaire (PSQ) [32]. These scales captured the user’s perception of the device through questions scored on a scale from 0 to 5 based on the level of satisfaction experienced. Furthermore, participants completed the SF-12 quality of life questionnaire after the final session [33].

Additionally, all therapists completed the QUEST 2.0 questionnaire and a satisfaction questionnaire specifically designed for physiotherapists [34].

### 2.5. Data Analysis

Descriptive statistics were utilized to summarize the quantitative data, employing mean and standard deviation (mean ± standard deviation) for parametric data and median and interquartile range (IQR) for non-parametric data. To determine the distribution type of the sample, a Shapiro–Wilk test (n < 30) or a Kolmogorov–Smirnov test (n > 30) was conducted, alongside Q-Q plots and histograms. In cases where the assumptions of parametric statistics were not met, a paired t-test or a Wilcoxon signed-rank test was employed for evaluating pre- and post-session measurements. A significance level of α = 0.05 was set, and differences were considered statistically significant when *p* < 0.05. Additionally, Cohen’s d effect size measure was calculated to assess the strength of the effect based on Cohen’s benchmarks (d = 0.2 is small, d = 0.5 is medium, and d = 0.8 is considered a large effect size) [35].

For intersession evaluations, one-way repeated measures ANOVA was conducted to compare the means of different assessments within the same group over time. All measures were treated as independent, and the data satisfied the criteria of normality and homoscedasticity. The sphericity criterion was assessed using the Mauchly test, and corrective measures were employed if the criteria were not met. Adjustments to the numerator and denominator degrees of freedom were made by multiplying the adjustment factor ε (epsilon) for effect tests. The choice of epsilon guideline was determined by the Greenhouse–Geisser epsilon, with a value of 0.75. If the value exceeded 0.75, the Huynh–Feldt epsilon was applied; otherwise, the Greenhouse–Geisser epsilon was used. The reporting of ANOVA results adhered to APA standards, including the F-statistic, degrees of freedom, significance level, effect size, and statistical power (β-1). Following the establishment of mean differences, post hoc rank tests were conducted to identify which means were significantly different. The Bonferroni test was employed, comparing means after rejecting the null hypothesis of equal means with the ANOVA test. The mean, standard deviation, and 95% confidence intervals of sample pairs with statistically significant differences (*p* < 0.05) were reported [36].

Furthermore, a correlation analysis was conducted to examine the associations between dependent variables (questionnaires) and independent variables (demographic information, anthropometric data, etc.). Pearson’s correlation was used for parametric variables, while Spearman’s correlation was employed for non-parametric variables. When one of the variables to be correlated was non-parametric, Spearman’s correlation was used. Correlation coefficients ranging from 0.00 to 0.30 were interpreted as poor, coefficients from 0.30 to 0.70 as moderate, and coefficients of 0.70 or higher as excellent. The significance level was set at 0.05 [37].

All analyses and graphical representations were performed using RStudio^®^ version 2022.7.2.576 (RStudio, PBC, Boston, MA, USA), IBM^®^ SPSS^®^ Statistics v29 software (IBM Corporation, Armonk, NY, USA), and Microsoft Excel^®^ 2019 (Microsoft Corporation, Redmond, WA, USA).

## 3. Results

Fourteen participants with ABI were enrolled in the study. One participant was excluded after screening due to exceeding the maximum weight limit. The participants had a mean age of 42.0 ± 10.8 years, a mean weight of 70.2 ± 14.9 kg, and a mean height of 170.2 ± 9.0 cm. Comprehensive information regarding their anthropometric measurements is provided in Table 1. All participants remained in the study until the last visit; thus, there were no dropouts. Regarding therapists involved in the study, there were three who had a mean age of 38.0 ± 9.5 years, with 12.3 ± 8.3 years of experience in neurorehabilitation and 16.0 ± 9.1 years of overall rehabilitation experience.

**Figure 2:**
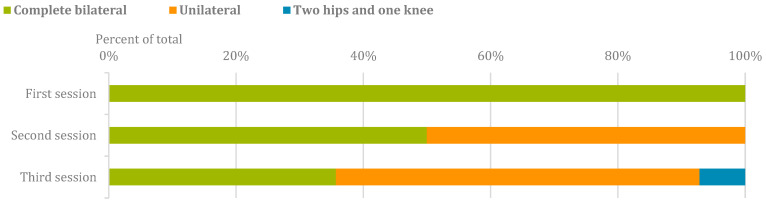
Distribution of modular configurations across sessions.

The device was used in 42 sessions overall. Figure 2 shows the STELO configurations that were utilized during the sessions. It is worth noting that, in the first session, all participants used the system in a complete bilateral configuration as per protocol. In sessions two and three, new module configurations were introduced based on the participants’ needs. Nine participants (64.3%) tried at least two different module configurations during the study.

No SAEs were observed throughout the study and no falls were reported involving either therapists or participants. Moreover, no physical injuries were noted during the sessions. Spasticity assessment before and after the use of the device showed no increase, with a median of 0 (IQR = 0) according to the MAS scale at baseline and at the end of the session. Statistically significant differences were obtained when comparing heart rate and systolic blood pressure before and after, both with a small effect size (d = 0.3). Detailed information on vital signs before and after STELO intervention can be found in Table 2.

**Figure 3:**
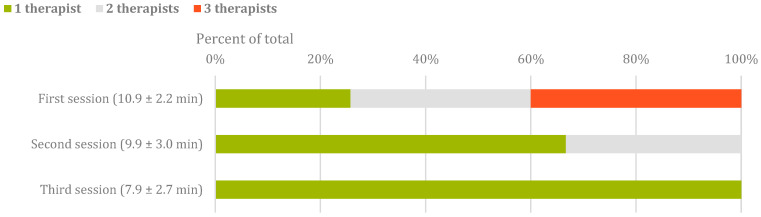
Assistance during donning the device in the three sessions. The time indicated in parentheses is the average time spent donning in that session.

The average device adjustment time was 9.8 ± 2.9 min throughout the study. Statistically significant differences were found in the times required for device adjustment across the three sessions: F (2) = 5.4, *p* = 0.01, η^2^ = 0.3, and β-1 = 0.8. Specifically, the scores in session 3 (7.9 ± 2.7 min) were lower than those in session 1 (10.9 ± 2.2, *p* = 0.05 [95% CI −5.9, 0.0]) and session 2 (9.9 ± 3.0, *p* = 0.05 [95% CI −4.1, 0.0]). The average device removal time was 2.2 ± 0.8 min. The level of assistance required to adjust the device to the participants is shown in Figure 3. Significant correlations with strong associations (r = 0.6) were found between the time required for adjustment and participants’ weight, hip width, and shoe size. In addition, there was a significant correlation between the number of different STELO configurations used by the participants and the participants’ FAC (r = 0.5, *p* = 0.04).

The mean walking time with the device was 21.2 ± 7.1 min. The average time required to complete the 10 mwt was 117.8 ± 74.3 s, and the average distance covered during the 6 mwt test was 29.9 ± 15.8 m. A progression in the distance covered with the sessions in the 6 mwt and a decrease in the time required for the 10 mwt were observed. Session-wise results can be visualized in Table 3. However, there were no statistically significant differences in the distance walked during the 6 mwt or the time taken to complete the 10 mwt across sessions.

**Figure 4:**
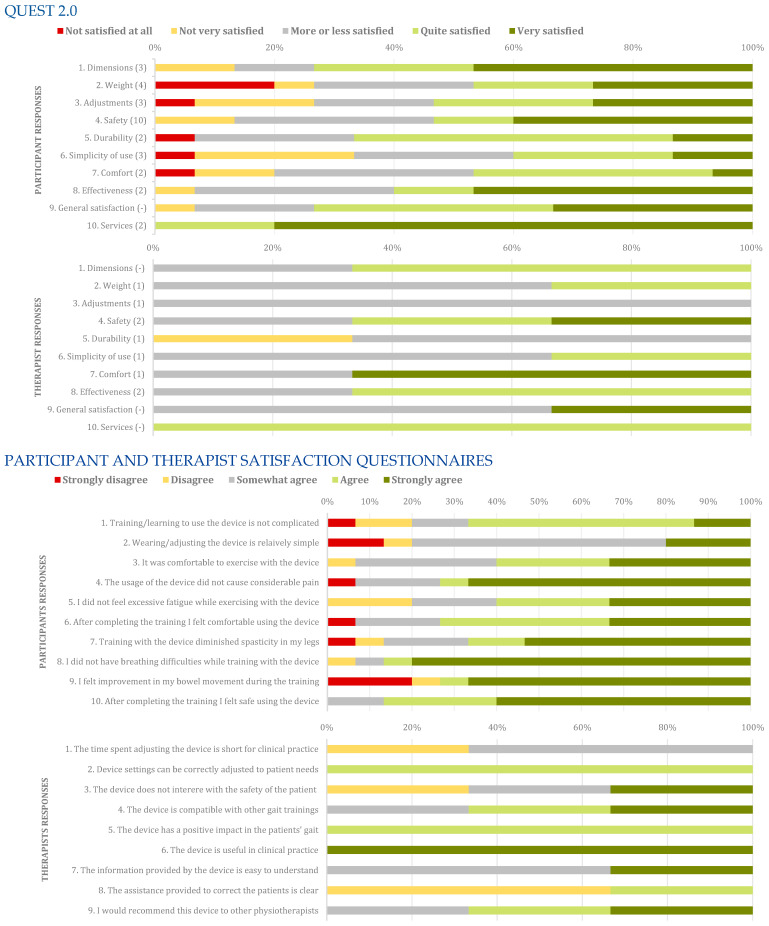
Satisfaction levels of participants and therapist in participants’ satisfaction questionnaire, physiotherapists’ satisfaction questionnaire, and QUEST 2.0. The number in brackets in Y axis of QUEST 2.0 represents how frequent an item was indicated within the top 3 most important items of the QUEST by the participants.

In relation to participant satisfaction data based on the QUEST 2.0 scale, the median score per item was 4 out of 5 possible points (IQR = 2), with a median scale score of 38 out of 50 (IQR = 8). Similarly, for the patients’ satisfaction questionnaire, the median score per item was 4 out of 5 (IQR = 2) and the scale score was 40 out of 50 points (IQR = 8). Regarding the satisfaction perceived by therapists, the median score per item was 3 out of 5 points (IQR = 1). The median score on the QUEST 2.0 scale reported by therapists was 36 out of 50 points (IQR = 3). In the physiotherapists’ satisfaction questionnaire, the median score per item was 4 out of 5 points (IQR = 2). The overall median score on the physiotherapists’ satisfaction questionnaire scale was 33 out of 45 points (IQR = 3). Safety was considered the item most important by participants and therapists. The results by item and items considered most important by participants and therapists can be found in Figure 4.

Significant correlations with strong associations (r = 0.6, *p* = 0.01) were found between perceived ease-to-use by participants and MCS-12. Furthermore, a significant strong association (r = 0.5, *p* = 0.04) was also found between the total score of the patients’ satisfaction questionnaire and the PCS-12 component.

Age showed a significant negative association with overall self-perceived satisfaction in QUEST 2.0 (rho = −0.7, *p* = 0.006). Duration of ABI also showed strong negative associations with ease-to-use (rho = −0.6, *p* = 0.02) and perceived ease of adjustment (rho = −0.7, *p* = 0.004). Participant weight exhibited strong negative associations with comfort after device use (rho = −0.07, *p* = 0.005) and safety after completing training (rho = −0.06, *p* = 0.01). Hip width of participants also displayed significant negative associations with comfort after device use (rho = −0.8, *p* < 0.001) and safety after completing training (rho = −0.7, *p* = 0.01). Femur length correlated negatively with perception of device dimensions (rho = −0.5, *p* = 0.04). Calf circumference was negatively associated with comfort after completing training (rho = −0.7, *p* = 0.006). Torso width was related to comfort after completing training (rho = −0.7, *p* = 0.004) and safety after completing training (rho = −0.5, *p* = 0.005). Foot size positively correlated with perceived weight by participants (rho = 0.5, *p* = 0.04) and negatively correlated with overall satisfaction (rho = −0.7, *p* = 0.006) and safety after completing training (rho = −0.7, *p* = 0.002). TUG time positively correlated with self-perceived comfort (rho = 0.6, *p* = 0.003) and improvements in bowel function (rho = 0.5, *p* = 0.04). The time taken for adjustment of the device was negatively related to ease-to-use (rho = −0.7, *p* = 0.007) and safety when completing training (rho = −0.5, *p* = 0.04). Ease of adjustment was related to the number of different exoskeleton configurations used (d = 0.5, *p* = 0.04). Results between both questionnaires were positively correlated.

Regarding the therapists’ questionnaires, negative correlations (rho = 1.0, *p* < 0.001) were found between the year of birth and the overall score of QUEST and the physiotherapists’ satisfaction questionnaire. On the other hand, positive correlations were found between experience in neurological rehabilitation (rho = 1.0, *p* < 0.001) and the overall score of both questionnaires. No significant correlation was obtained between experience in the use of robotics and satisfaction with the device.

## 4. Discussion

The present study aimed to conduct initial tests on patients using the first fully modular robotic gait device with functional autonomy in its joints. When all the joints are physically connected, they collaborate synchronously. Additionally, it is possible to attach and detach joints to adapt to the gait and functionality of each specific patient. This novel concept has several clinical and economic benefits that can enhance the usability of gait training devices. Therefore, this study sought to investigate the feasibility of functional modularity applied to robotic gait devices through a series of sessions involving participants diagnosed with ABI. Usability was evaluated through the presence of AEs and questionnaires assessing self-perceived satisfaction by participants and therapists. Moreover, potential correlations between perceived satisfaction and different clinical data were explored.

Fourteen ABI patients participated in the study, where different sets of modules were tested to adapt to each participant’s functionality, except for the first session, in which the device was always used in its complete bilateral configuration to test it. After the bilateral configuration, the unilateral configuration was the most used among the participants, since most of them presented hemiplegia, as it is common in persons with ABI. Additionally, one participant used the unilateral configuration along with the contralateral hip module, as this configuration suited his impairment. Participants who exclusively tested the bilateral configuration did so due to their functional impairment preventing them from utilizing different configurations. No other module configurations were used because they did not meet the needs of the participants included in the study.

The assistance required for donning the STELO varied during the sessions. This was because the therapists were not trained in the use of the device, so they needed practice in the early sessions, hence the need for multiple therapists. In the subsequent sessions, the assistance required decreased to the point where it could be carried out by a single therapist. Nevertheless, the time required to adjust the device to the patient decreased considerably, despite having less assistance, as shown in Figure 3. Additionally, several participants were in the subacute period of their ABI, which could also increase the time required for donning the device as their mobility was severely limited. The literature shows that a patient should be able to independently adjust the robotic device in less than 5 min [38]. However, other studies have shown that the current average time required for adjustment is 9 min and 4 s. The mean time required to adjust the device during our study was 9.8 ± 2.9 min [38,39,40]; however, this time was reduced as the sessions progressed, reaching 7.9 ± 2.7 min during the last set of sessions. Therefore, the donning time for STELO showed to be below the average. Anyway, it has been shown that therapists must be trained on device adjustment ahead of device implementation with patients in order to reduce donning time and to increase session effectiveness.

The most commonly used questionnaires to assess patient and therapist satisfaction with robotic gait devices currently are QUEST 2.0 and the participant satisfaction questionnaire [11]. The median score obtained by participants using the STELO in the QUEST 2.0 questionnaire was 38 out of 50 (IQR = 8), resulting in a 78.0% percentage score relative to the maximum possible score. The participant satisfaction questionnaire yielded an 80% score. In a population similar to the one evaluated in this study, for individuals with stroke, the Indego ^TM^ device (Parker Hannifin Corporation, Human & Control, Macedonia, OH, USA) obtained a normalized score of 71.2% in the participant satisfaction questionnaire after 16 sessions of device use [41]. Another satisfaction questionnaire yielded a score of 82.0% for the Exowalk (HMH Corp., Daejeon, Republic of Korea) after 20 sessions [42]. Regarding robotic devices that assist a single joint, the ankle robotic system ReStore (ReWalk Robotics, Inc., Marlborough, MA, USA) obtained a normalized score of 84.5% in the QUEST 2.0 after eight sessions [43], significantly higher than the perceived satisfaction with the T-FLEX, which obtained a score of 33.3% after one session [44]. The knee robotic device MAK (Marsi Bionics SL, Madrid, Spain) obtained a normalized score of 56% after three sessions [23] and 66.5% after nine sessions [22], while the knee robotic system ALLOR obtained a score of 84.0% after a single session [45].

Among these robotic gait training devices in spinal cord injury (SCI), the ReWalk (ReWalk Robotics, Inc., Marlborough, MA, USA) is the only one that has reported satisfaction with the use of such a device in community settings for individuals with SCI, resulting in a normalized score of 74.0% in this type of environment [46]. In clinical settings, it showed a normalized score of 42.0% in the participant satisfaction questionnaire [47] and 65.2% in a different type of questionnaire not reflected here [48]. In individuals with SCI, the ABLE device (ABLE Human Motion, Barcelona, Spain) obtained a median score of 33.0 (IQR = 7.5) out of 40 in QUEST 2.0 after 12 sessions of use, resulting in a normalized score of 82.5% relative to its maximum possible score [49], like the score achieved by the Kinesis device in the same population [50]. The H2 system showed a normalized score of 67.8% in QUEST 2.0 in this population [51].

Regarding therapists’ satisfaction with robotic devices for gait assistance, a review found only three studies that evaluated this variable among therapists [11]. It is worth mentioning that evaluating therapists’ satisfaction is essential because, as of today, these devices are mostly used for medical purposes in rehabilitation. Two of these studies assessed satisfaction using the therapists’ satisfaction questionnaire, obtaining normalized scores of 74.9% for the ReStore ankle device in stroke patients [43] and 85.5% for the Ekso device in individuals with multiple sclerosis [34]. In our study, we obtained a normalized score of 73.3% in the same questionnaire, similar to the ReStore device. It is important to note that this is the first time that this type of modular device has been tested in patients in a clinical trial; therefore, it is currently a prototype, unlike the previous ones that are considered marketable medical products. On the other hand, another study showed that there is a difference in satisfaction as the duration of device usage increases, resulting in higher satisfaction over time [52]. This finding may also explain the lower satisfaction among therapists in the present study, as only three sessions were conducted for each participant.

The findings from these studies suggest varying levels of satisfaction across different robotic gait training devices models/prototypes and populations. It is worth noting that factors such as disease, duration of use, rehabilitation goals, questionnaire used, and individual characteristics of the patient can influence the perceived satisfaction with these devices. Related to this, a study showed significant relationships between the number of sessions performed and the overall satisfaction and efficacy perceived by patients [34]. Further research and larger-scale studies are necessary to gain a more comprehensive understanding of the satisfaction levels associated with different robotic devices in neurorehabilitation settings.

Our results reveal a significant reduction in systolic blood pressure following the use of the device. This phenomenon is consistent with the commonly observed decrease in blood pressure levels after performing physical exercise, as previously documented in the scientific literature [53]. However, diastolic blood pressure showed no statistically significant changes. Regarding heart rate and oxygen saturation, there was an average increase after device application, although this increase did not reach statistical significance.

Regarding walking tests, statistical significance was not reached, despite an observed gradual improvement. This progress may be linked to the learning curve of device usage. However, caution is advised in extrapolating these findings to clinical efficacy due to the limited number of sessions. The variability in the 10 mwt between the first and second session may be attributed to inherent participant differences, reflecting the diverse nature of neurological pathologies. While subsequent sessions showed a potential adaptation, substantial standard deviations, especially in the first session, emphasized a participant variability probably tied to diverse neurological conditions. Although promising, the small sample size and short duration of this study require careful interpretation, highlighting the need for further research with a longer intervention period to validate these initial observations and assess sustained device impact.

This study has several limitations. Firstly, the number of sessions may be limited to evaluate satisfaction with the device. However, these results will serve as a baseline for comparison with subsequent studies where optimizations are implemented in the device, allowing testing for an increase in satisfaction following those changes. Additionally, it would be interesting to compare perceived satisfaction across different robotic gait devices in future research. Such a comparison would be more realistic as it is related to the same type of intervention, duration, intensity, professionals, and so on. Furthermore, comparing conventional rehabilitation interventions with interventions involving robotic devices would be valuable, as the level of satisfaction and motivation of each patient with their therapy is crucial for achieving good outcomes. However, it should be noted that the questionnaires used in this study are specific to technological devices. Therefore, another type of questionnaire should be used to facilitate comparisons between conventional and robotic gait training rehabilitation.

For future studies, the following recommendations are suggested. Firstly, increasing the number of sessions to assess long-term satisfaction with the device would provide more robust data. Secondly, incorporating user feedback and suggestions for device improvements could enhance overall satisfaction and engagement. Additionally, conducting a comparative study between different gait assistive devices under similar intervention conditions would provide valuable insights. Lastly, utilizing standardized questionnaires that allow for direct comparison between conventional and robotic rehabilitation could help evaluate the efficacy and satisfaction of different intervention approaches.

## 5. Conclusions

STELO, the modular robotic gait device, proved to be safe for both professionals and patients with ABI. Its usability was also considered favorable for both as the adjustment time was shorter compared with other similar devices. Furthermore, participants and therapists expressed a high level of satisfaction with the device. Due to the limited number of sessions, no significant improvements were observed in gait tests with the device, so further research involving longer interventions should be conducted in order to investigate the efficacy of this device.

## Figures and Tables

**Table 1:** Sample description.

P	Sex	Age	Cause	Mobility	Months Since ABI	Weight (kg)	Heigth (cm)	TUG	PCS-12	MCS-12	FAC
1	Male	50	Stroke	Cane + AFO	13.7	85	180	33.1	34.0	53.4	3
2	Female	39	Stroke	Wheelchair	15.5	52	168	22.0	27.4	56.7	2
3	Male	46	Stroke	Wheelchair	24.4	94	167	56.5	39.6	60.8	2
4	Female	50	Stroke	Assistive device	18.7	80	167	114.7	34.2	36.4	3
5	Female	35	Stroke	Wheelchair	18.3	51	163	25.9	38.5	57.8	3
6	Female	23	Stroke	Autonomous	19.0	53	161	14.3	41.0	56.1	4
7	Female	27	Tumor	Wheelchair	18.0	50	154	31.0	45.7	45.5	2
8	Female	46	Stroke	Assistive device	30.4	63	161	15.7	36.2	38.1	2
9	Male	38	TBI	Wheelchair	8.1	85	180	43.6	56.6	43.5	1
10	Male	48	TBI	Assistive device	8.1	74	169	49.0	53.6	60.8	3
11	Male	31	TBI	Assistive device	29.8	65	173	36.3	40.9	32.0	1
12	Male	58	Stroke	Wheelchair	161.3	68	178	44.6	40.5	56.8	3
13	Male	39	TBI	Wheelchair	221.5	87	186	20.3	54.4	48.0	1
14	Male	59	Stroke	Wheelchair	136.0	77	176	42.5	30.2	62.9	3

P—participant, AFO—ankle foot orthosis, TBI—traumatic brain injury, ABI—acquired brain injury, TUG—timed up and go test, PCS-12—physical score of SF-12, MCS—mental score of SF-12, FAC—functional ambulation categories.

**Table 2:** Vital signs before and after device use.

*Vital Sign*	Start	End
**Oxygen saturation (%O_2_)**	96.2 ± 2.3	96.6 ± 1.5
Heart rate (beats/min) **	80.4 ± 16.0	87.1 ± 20.5
Systolic blood pressure (mmHg) *	117.2 ± 11.9	117.1 ± 11.1
**Diastolic blood pressure (mmHg)**	75.4 ± 9.8	75.8 ± 9.8

mmHg—millimeters of mercury, * denotes *p* < 0.01 and ** denotes *p* < 0.001.

**Table 3:** 10 mwt and 6 mwt across sessions.

Session	10 mwt (s)	6 mwt (m)
**1**	142.4 ± 105.9	24.2 ± 13.4
**2**	105.4 ± 63.2	31.3 ± 13.3
**3**	110.3 ± 76.9	32.8 ± 18.8

10 mwt—ten-meter walk test. 6 mwt—six-minute walking test.

## Data Availability

Marsi Bionics SL will provide access to de-identified patient level data that underly the results in this article in response to scientifically valid research proposals. Marsi Bionics SL will consider requests from qualified researchers for access to the data. Marsi Bionics will make reasonable efforts to fulfill all data requests for legitimate research purposes, but there might be instances in which retrieval or delivery of data is not feasible, such as those involving, for example, patient privacy, requirements for permissions, contractual obligations, and conflicts of interest. All those receiving access to data will be required to enter into a data use agreement provided by Marsi Bionics SL, which will contain the terms under which the data will be provided.
